# Serological Markers of Selected Sexually Transmitted Infections (HBV, HCV, HIV, and Syphilis) in Patients with Anogenital Warts: A Two-Centre, Five-Year Study

**DOI:** 10.3390/pathogens15101062

**Published:** 2026-10-08

**Authors:** Aziz Alper Biten, Meryem Çetin, Simge Süel Eroğlu, Ömer Kutlu, Hakan Şıvgın, Şirin Çetin

**Affiliations:** 1 General Directorate of EU and Foreign Affairs, Ministry of Health, Ankara 06800, Türkiye; alper.biten@saglik.gov.tr; 2 Department of Medical Microbiology, Amasya University Sabuncuoğlu Şerefeddin Training and Research Hospital, Amasya 05200, Türkiye; meryem.cetin@amasya.edu.tr; 3 Department of Dermatology and Venereology, Amasya University Sabuncuoğlu Şerefeddin Training and Research Hospital, Amasya 05200, Türkiye; simgesuel24@gmail.com; 4 Department of Dermatology and Venereology, Tokat Gaziosmanpaşa University Faculty of Medicine, Tokat 60250, Türkiye; omerkutlu22@gmail.com; 5 Departmant of Internal Medicine, Tokat Gaziosmanpaşa University Faculty of Medicine, Tokat 60250, Türkiye; sivginhakan@gmail.com; 6 Department of Biostatistics, Amasya University Sabuncuoğlu Şerefeddin Training and Research Hospital, Amasya 05200, Türkiye

**Keywords:** anogenital warts, human papillomavirus, sexually transmitted infections, seroprevalence, hepatitis B, syphilis, serological screening

## Abstract

**Background:** Human papillomavirus-induced disruption of the anogenital mucosal barrier may increase susceptibility to other sexually transmitted infections (STIs), and guidelines recommend STI screening in patients with anogenital warts (AGW). We aimed to determine the seroprevalence of concomitant STIs in patients with AGW over a five-year period and to perform an exploratory assessment of associated demographic factors. **Methods:** In this retrospective, cross-sectional study, all consecutive patients clinically diagnosed with AGW between December 2020 and October 2025 (*n* = 779) were evaluated. Serological results for HBsAg, anti-HBs, anti-HCV, anti-HIV, and syphilis (treponemal and non-treponemal) were obtained. Seroprevalence was estimated with Wilson 95% confidence intervals (CIs) using the number of patients actually tested as the denominator. Patterns of multiple reactive serological markers were described, and the relationship between age and seropositivity was examined using a Cochran–Armitage trend test. Associations of demographic factors with concomitant STI seropositivity were explored using Fisher’s exact test with Woolf logit CIs and a complementary Firth penalized logistic regression; given the small number of events, these analyses were considered exploratory and hypothesis-generating. **Results:** Among 779 patients (585 [75%] male; mean age, 35.80 ± 12.32 years), the overall seroprevalence of concomitant STIs was 2.4% (19/779; 95% CI, 1.6–3.8). Seroprevalence was 1.4% for HBsAg, 0.9% for anti-HCV, 0.2% for anti-HIV, and 1.6% for treponemal antibodies, while anti-HBs immunity was present in 53.5% of patients. Two patients (10.5% of seropositive cases) had more than one concomitant STI. Seropositivity increased from 0.7% in patients aged 17–24 years to 3.5% in those aged ≥45 years, although the trend was not statistically significant (*p* = 0.13). Anti-HBs negativity was associated with concomitant STI seropositivity (OR = 5.63; 95% CI, 1.60–19.83; *p* = 0.003); this association was attenuated and no longer statistically significant after excluding HBV markers (OR = 2.73; 95% CI, 0.70–10.69; *p* = 0.200). A complete-case analysis restricted to patients with a fully defined testing panel yielded a similar prevalence estimate (2.8%; 95% CI, 1.7–4.5). **Conclusions:** The seroprevalence of the tested markers in patients with AGW was low but non-negligible and increased with age, while the subgroup of markers more consistent with current infection was less common. Anti-HBs negativity was associated with the composite outcome, but a sensitivity analysis showed this was largely attributable to its expected link with HBV status. These findings support guideline-recommended serological screening—including hepatitis B vaccination assessment—in all patients with AGW, and should be regarded as hypothesis-generating given the limited number of seropositive cases.

## 1. Introduction

Anogenital human papillomavirus (HPV) infection is the most common sexually transmitted infection (STI) worldwide and manifests clinically as anogenital warts (AGW). Although HPV types 6 and 11 cause mostly benign AGW, oncogenic types may co-circulate [1,2,3,4]. From a microbiological standpoint, the importance of AGW extends beyond the lesions themselves: HPV-related disruption of the stratified squamous epithelium and local immune modulation compromise the anogenital mucosal barrier and, together with shared transmission routes, may increase susceptibility to other sexually transmitted pathogens, including HIV, hepatitis B virus (HBV), hepatitis C virus (HCV), and Treponema pallidum [5,6,7,8,9,10]. Accordingly, current guidelines recommend serological screening for concomitant STIs in patients presenting with AGW [5].

HPV types 6 and 11 are low-risk, non-oncogenic genotypes that together account for the great majority of AGW lesions worldwide, typically identified in more than 90% of histologically or molecularly confirmed cases [1,2,3,4]. These genotypes preferentially infect the stratified squamous and transitional epithelium of the anogenital tract, where episomal viral replication and E6/E7-mediated disruption of keratinocyte cell-cycle control drive the characteristic exophytic, hyperproliferative wart morphology, in contrast to the integrative, transformation-associated pattern seen with high-risk oncogenic genotypes such as HPV16/18. Co-infection with oncogenic HPV types has nonetheless been reported in a subset of AGW lesions, and validated genotyping methods (e.g., the Linear Array HPV Genotyping assay) have been used to characterize type-specific HPV distribution in other at-risk populations, including HIV-positive individuals [11]. In the present study, AGW was diagnosed clinically and lesions were not routinely genotyped (Section 2.2); consequently, we cannot report the type-specific distribution of HPV in our cohort, and our seroprevalence findings should be interpreted as pertaining to clinically diagnosed AGW irrespective of the underlying genotype.

Several studies have examined serological markers of concomitant STIs in patients with AGW, with reported rates for HBsAg, anti-HCV, anti-HIV, and syphilis varying widely across populations, testing panels, and case definitions [7,8,9,12,13]. Most of these reports, however, are drawn from a single centre, cover short observation periods, or do not distinguish markers of current infection from serological evidence of previous exposure or persistent serological reactivity—a distinction with direct implications for patient counselling and public-health risk assessment. In Türkiye specifically, published data on this topic remain limited to a small number of single-centre series [7,8,9,12], and none, to our knowledge, has combined a multi-year observation period with a two-centre design or reported age-stratified trends alongside exploratory demographic associations.

The reported burden of AGW varies widely; a 2025 systematic review of 187 studies from 49 countries reported prevalence estimates of 0.5–33.1% [14], and surveillance data indicate a rising incidence of viral STIs in several regions [6]. However, region-specific data on the seroprevalence of concomitant serological findings among patients with AGW remain limited, and the demographic factors associated with concomitant STI seropositivity are incompletely characterized.

Against this background, we performed a five-year, two-centre cross-sectional study to characterize the seroprevalence of concomitant sexually transmitted infections (hepatitis B, hepatitis C, HIV, and syphilis) in patients with AGW, with prevalence estimates reported alongside 95% confidence intervals. As a secondary, exploratory objective, we examined demographic factors potentially associated with concomitant STI seropositivity. Given the limited number of seropositive cases, this analysis was conceived as hypothesis-generating, and no clinical prediction rule was derived. To our knowledge, this is among the few two-centre, multi-year seroprevalence studies of concomitant STIs in patients with AGW conducted in Türkiye, and the first to include the Central Black Sea region, thereby extending previous single-centre reports to a larger and geographically broader population [7,8,9,12]. Specifically, we sought to characterize the serological findings and to explore their associations with demographic factors, while acknowledging the limitations of serology in determining the clinical significance of individual reactive markers.

## 2. Materials and Methods

### 2.1. Study Design and Ethics

In this retrospective, cross-sectional study, we reviewed eligible patients aged ≥17 years clinically diagnosed by a dermatologist with AGW at two centres (Amasya University Sabuncuoğlu Şerefeddin Training and Research Hospital and Tokat Gaziosmanpaşa University Faculty of Medicine) between December 2020 and October 2025. The study complied with the Declaration of Helsinki and the Tokat Gaziosmanpaşa University Non-Interventional Clinical Research Ethics Committee (approval no. E-15235480-050.04-788985; decision 26-MOBAEK-351). The requirement for informed consent was waived owing to the retrospective design. Reporting follows the STROBE statement for cross-sectional studies (Supplementary File S1).

Patients were included if they had a clinical diagnosis of AGW recorded in the hospital information system within the study period and were aged ≥17 years; records with missing age or sex, or with a diagnosis date outside the study period, were excluded. No patients were excluded on the basis of missing serological results, which were instead handled using marker-specific denominators (Section 2.4). Repeated visits for the same patient were deduplicated, and each patient was included only once based on the index AGW presentation during the study period. The main clinical and epidemiological characteristics of the study population, including age, sex, and admission type, are summarized in Table 1. Data on HIV pre-exposure prophylaxis (PrEP) use were not available in the retrospective hospital records and could therefore not be incorporated into risk-profile or screening-practice analyses; this is acknowledged as a limitation (Section 5).

Patients underwent clinical evaluation by a dermatologist at the time of AGW presentation. Serological tests were ordered in accordance with the institution’s routine practice and the clinical assessment of the treating dermatologist, rather than based on a standardized, study-specific panel applied uniformly to all patients.

Consequently, the availability of individual serological markers varied among patients. When syphilis testing was performed, the laboratory workflow used a reverse-sequence algorithm: a treponemal antibody test was used for initial screening, reactive samples were followed by a quantitative non-treponemal test (VDRL/RPR), and discordant treponemal-reactive/non-treponemal-non-reactive results were further evaluated with TPHA when available. Internal positive and negative controls were included in each analytical run, and laboratory quality was monitored through external quality assessment/proficiency testing procedures.

The diagnosis of AGW was established clinically by the dermatologist based on characteristic visual examination findings, consistent with routine clinical practice at both centres. Histopathological examination was performed when clinically indicated—specifically for lesions exhibiting atypical features or cases requiring differential diagnosis from other verrucous lesions. HPV genotyping was not performed as part of the study protocol. Testing for Neisseria gonorrhoeae and Chlamydia trachomatis was not performed, as nucleic acid amplification testing for these pathogens was not part of the routine serological screening panel ordered for AGW patients at either study site during the study period; this is discussed further as a limitation (Section 5).

### 2.2. Microbiological Methods

All serological assays were performed in the hospital’s accredited clinical microbiology laboratory using standardized platforms. HBsAg, anti-HBs, anti-HCV, and anti-HIV were determined by chemiluminescent/microparticle immunoassay (CMIA) performed on the Abbott ARCHITECT i2000SR automated immunoassay system (Abbott Laboratories, Abbott Park, IL, USA), according to the manufacturer’s instructions. Internal positive and negative controls were included in each analytical run. The laboratory also participated in an external quality assessment (proficiency testing) programme for the relevant serological assays, and test results were accepted only when predetermined internal and external quality control criteria were met.

Syphilis serology was performed using a reverse-sequence algorithm, in which a treponemal antibody test served as the initial screening test, followed by a quantitative non-treponemal test (VDRL/RPR) for reactive samples. Discordant results were further evaluated using TPHA as a second treponemal test.

### 2.3. Definitions

Concomitant STI seropositivity was defined as a positive result in at least one of HBsAg, anti-HCV, anti-HIV, or syphilis (reactive treponemal antibody and/or reactive VDRL/RPR/TPHA). Anti-HBs was analyzed separately as a marker of immunity, not as a component of the outcome. The demographic factors examined were sex, age (continuous and dichotomized at ≥30 years), and anti-HBs status. The term concomitant refers to the detection of a reactive serological marker in the same patient during the study period and does not imply temporal concurrence with the AGW diagnosis or a causal association, as the interval between serological testing and AGW diagnosis could not be established for all patients (Section 5). As a complementary analysis, a complete-case composite estimate was also calculated among patients with a fully defined testing panel (Section 3.2).

### 2.4. Statistical Analysis

Statistical analyses were performed using IBM SPSS Statistics version 26.0 (IBM Corp., Armonk, NY, USA) and the R statistical environment version 4.3 (R Foundation for Statistical Computing, Vienna, Austria). Two-sided *p* values < 0.05 were considered statistically significant. The distribution of continuous variables was assessed with the Shapiro–Wilk test and visual inspection of Q–Q plots; normally distributed variables were summarized as mean ± standard deviation (SD) and non-normally distributed variables as median (minimum–maximum), while categorical variables were expressed as frequency (percentage).

The seroprevalence of each serological marker was estimated as a proportion with Wilson score 95% confidence intervals (CIs), using the number of patients actually tested for each marker as the denominator to account for missing results. Group comparisons of continuous variables used the independent-samples *t*-test or the Mann–Whitney U test as appropriate, and categorical comparisons used the Pearson χ^2^ test or Fisher’s exact test when expected cell counts were below five. The trend in seropositivity across ordered age groups was evaluated with the Cochran–Armitage trend test, computed in R (package “DescTools”). To benchmark the cohort against national figures, the prevalence of each marker was compared with the corresponding general-population estimate for Türkiye using a two-sided one-proportion exact binomial test.

To mitigate small-sample and separation bias, a complementary multivariable Firth penalized logistic regression was fitted in R (package “logistf”). To address the potential structural overlap between anti-HBs negativity and the HBV component of the outcome, a prespecified sensitivity analysis repeated the association after excluding HBV markers from the composite outcome. The composite outcome was additionally disaggregated into a subgroup of markers potentially indicative of current infection (HBsAg positivity, anti-HIV reactivity, or dual treponemal-and-non-treponemal reactivity; clinical staging not confirmed) versus the full set of any reactive serological marker. Because only 19 seropositive events were observed, all association analyses were regarded as exploratory and hypothesis-generating, and no clinical prediction rule was derived. The pattern and extent of missing data were described for each marker.

## 3. Results

### 3.1. Patient Characteristics and Temporal Trend

**Figure 1:**
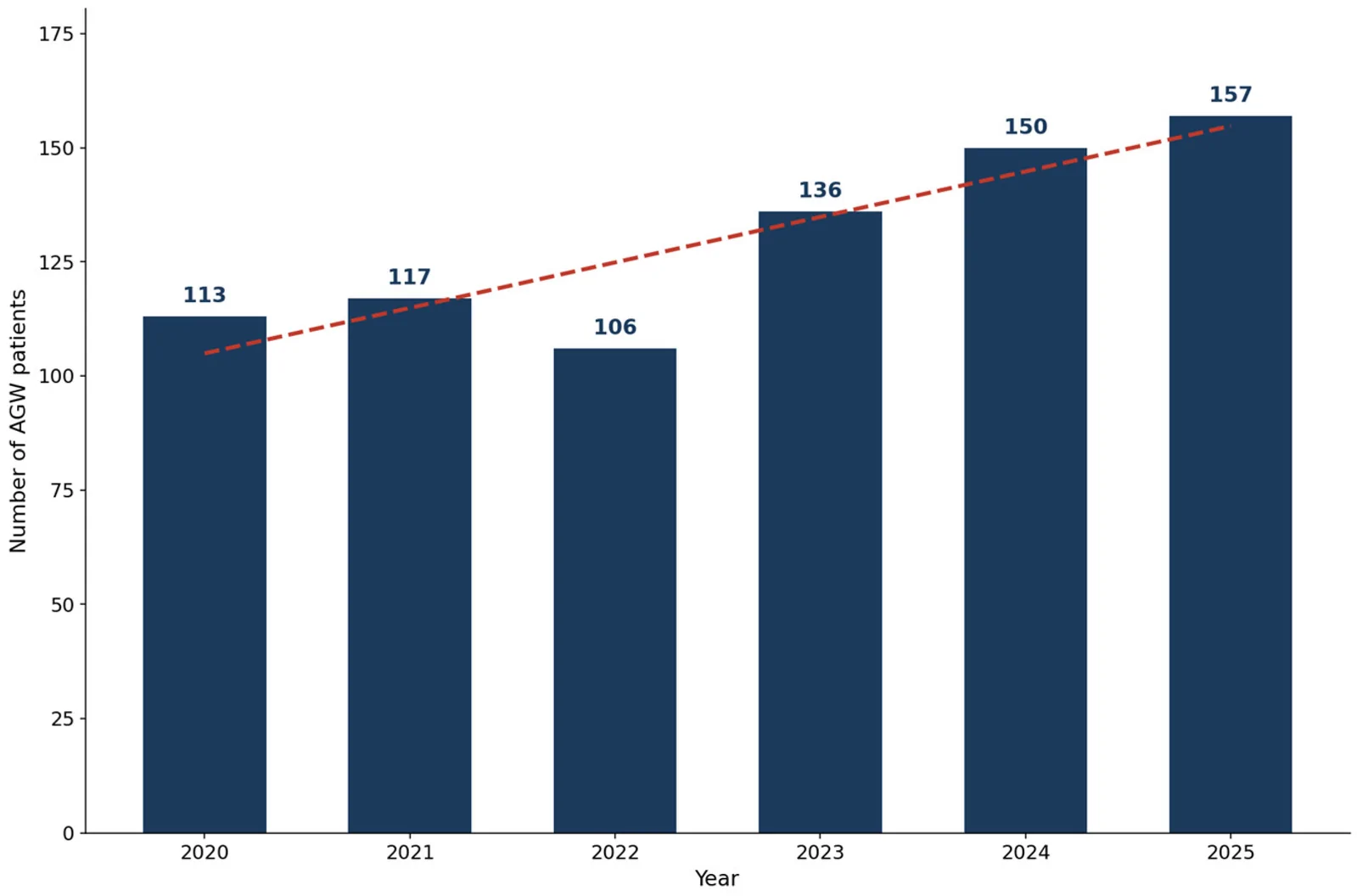
Annual number of patients presenting with anogenital warts, 2020–2025 (2020 and 2025 were partial years, whereas 2021–2024 represented full-year observation periods).

**Figure 2:**
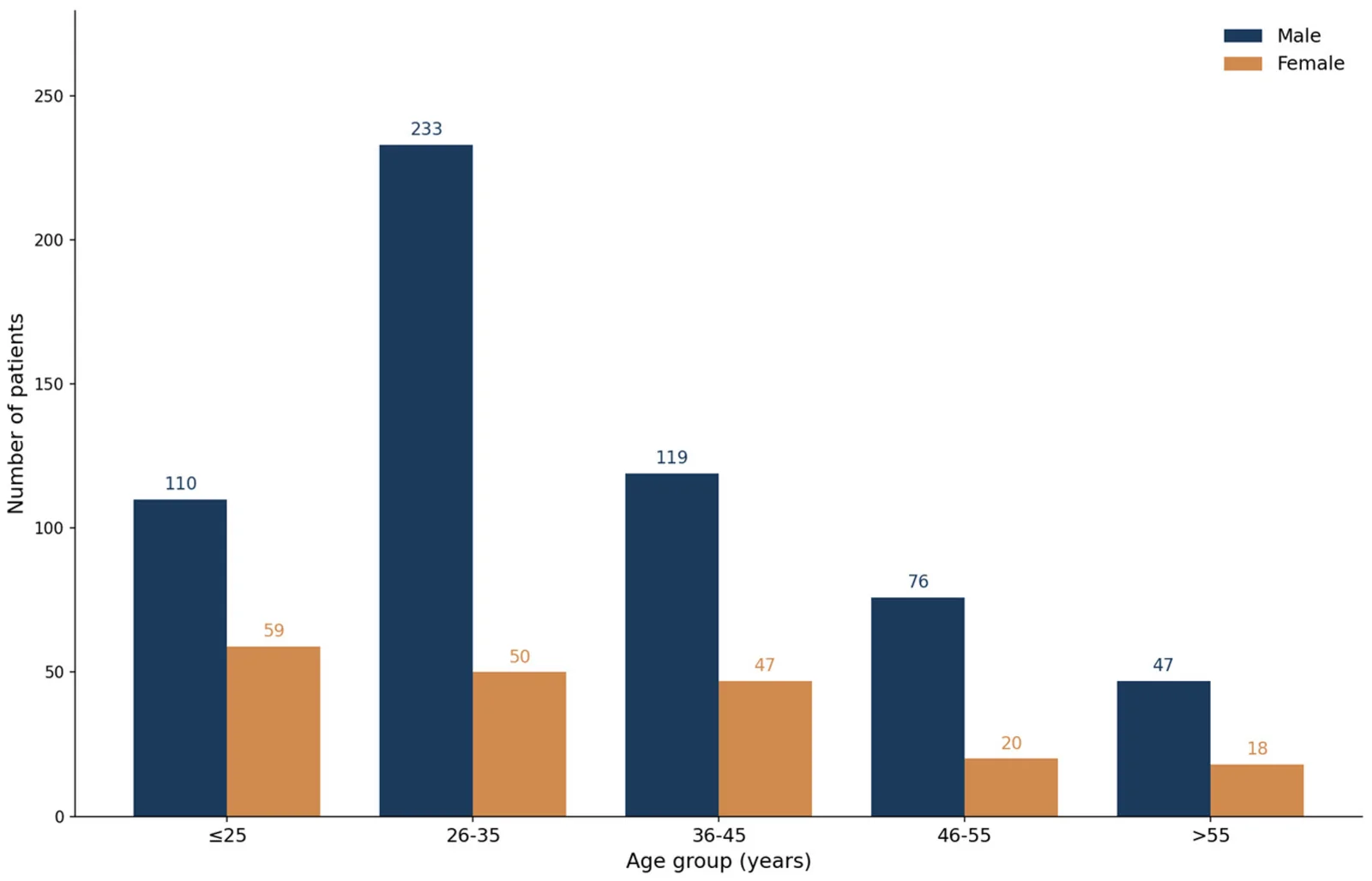
Age- and sex-specific distribution of patients with anogenital warts.

A total of 779 patients with AGW were included (Table 1): mean age 35.80 ± 12.32 years (range 17–87); 585 (75%) male. The number of AGW presentations rose from 113, 117, 106, 136, and 150 to 157 in 2020, 2021, 2022, 2023, 2024, and 2025, respectively (Figure 1). The age–sex distribution is shown in Figure 2; male patients clustered in the 26–35-year band.

### 3.2. Seroprevalence of Concomitant STIs

Seroprevalence estimates with Wilson 95% CIs are presented in Table 2. Any concomitant STI was detected in 19 patients (2.4%; 95% CI 1.6–3.8). HBsAg was positive in eight patients (1.4%) and anti-HCV in five patients (0.9%), anti-HIV was reactive in one patient (0.2%; 95% CI 0.0–0.9), and treponemal antibody was reactive in seven of 446 tested patients (1.6%). Anti-HBs was reactive (immune) in 53.5% (95% CI 49.3–57.7).

Because testing was incomplete and missing results were retained as missing rather than coded as negative, we performed complete-case sensitivity analyses. Table 3 shows the number of markers tested per patient: 171 (22.0%) had no marker tested and 421 (54.0%) had all five markers tested. Among 539 patients with all four routinely available markers tested, 15 had at least one reactive marker (2.8%; 95% CI, 1.7–4.5). Among 421 patients with all five markers tested, 12 had at least one reactive marker (2.9%; 95% CI, 1.6–4.9). These complete-case estimates were reported as sensitivity analyses.

### 3.3. Patterns of Multiple Reactive Serological Markers

Among patients with more than one reactive serological marker, two patients had combined anti-HCV and syphilis-related serological reactivity. These patterns are compatible with overlapping sexual and parenteral transmission routes, although transmission routes could not be established retrospectively.

### 3.4. Reactive-Marker Subgroups by Potential Clinical Relevance

Since HBsAg positivity, anti-HIV reactivity, and simultaneous reactivity in both treponemal and non-treponemal tests represent serological patterns in the more reactive-marker subgroup of potential clinical relevance, we defined this subgroup separately from the general group of patients with reactive markers—without classifying them as having confirmed active disease—even though clinical staging could not be retrospectively verified (Table 4). This subgroup consisted of 11 patients (1.4%; 95% CI: 0.8–2.5): eight HBsAg-positive patients, one anti-HIV-reactive patient, and two patients showing reactivity in both treponemal and non-treponemal tests.

Other patients with reactive markers fell into the following categories: anti-HCV reactivity (*n* = 5; active HCV infection could not be determined without HCV-RNA testing) and treponemal reactivity with a negative non-treponemal test (*n* = 4; clinical stage could not be determined without clinical and treatment history); a treponemal test subsequently became available for the previously indeterminate VDRL/RPR-reactive patient from the supplementary cohort, confirming dual reactivity and reclassifying that patient into the subgroup above. Simultaneous treponemal and non-treponemal reactivity is recognized as a characteristic serological pattern but does not, in itself, indicate disease activity or clinical stage. Anti-HCV reactivity indicates past exposure and may be consistent with active infection; however, active HCV infection cannot be confirmed without HCV-RNA testing. Treponemal reactivity observed alongside a negative non-treponemal test—when confirmed by a second treponemal test—is compatible with current or previous syphilis infection; however, the disease stage cannot be determined without clinical and treatment history.

Dual treponemal and non-treponemal reactivity is reported as a descriptive subgroup and does not by itself establish disease activity or clinical stage. Anti-HCV reactivity indicates prior exposure or possible current infection; without HCV-RNA testing, current HCV status could not be determined. Treponemal reactivity with a non-reactive non-treponemal test, when confirmed by a second treponemal assay, is compatible with current or previous syphilis infection; without clinical and treatment history, clinical stage and previous treatment status could not be established. CI, confidence interval.

### 3.5. Seropositivity by Age

**Figure 3:**
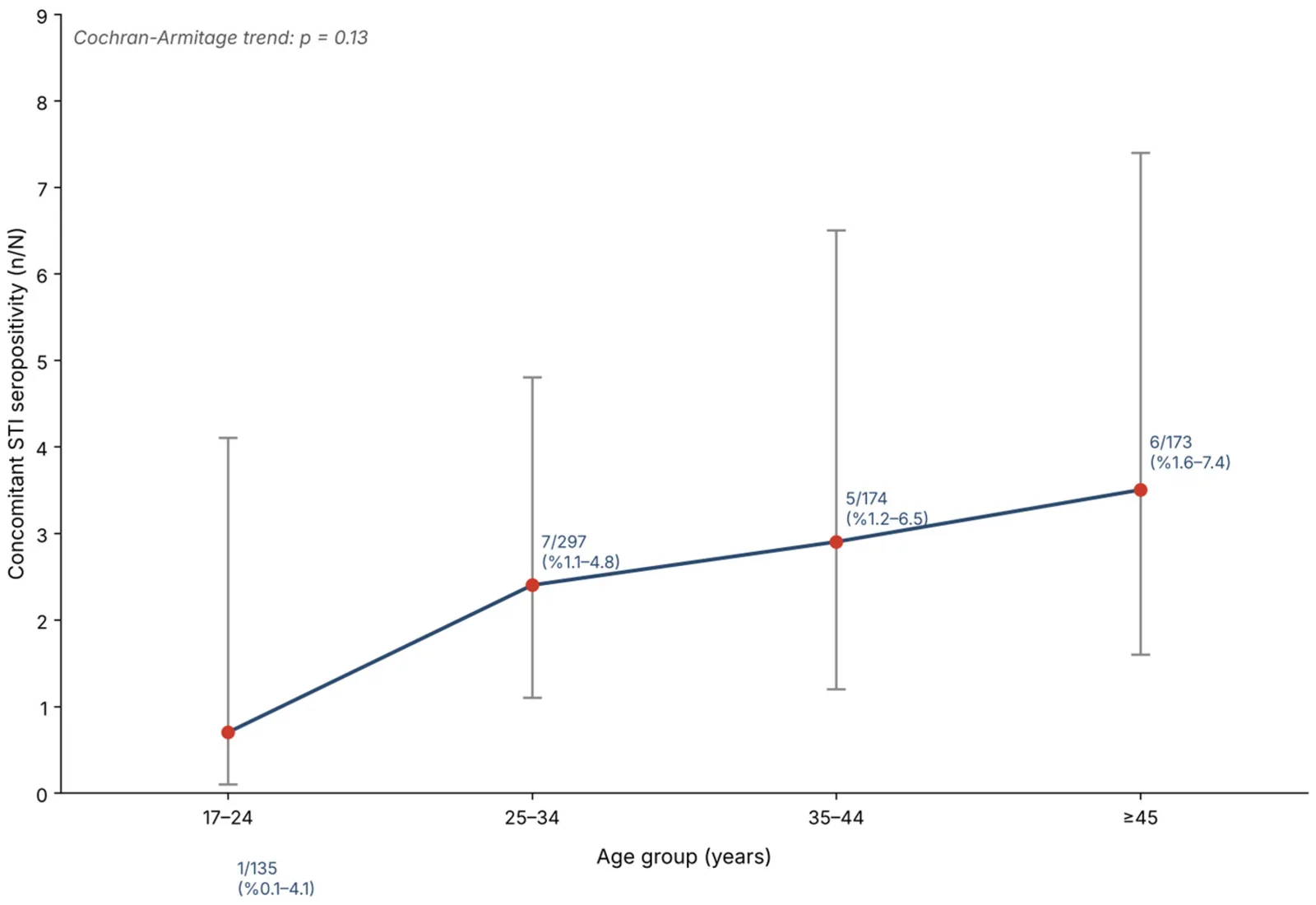
Concomitant STI seropositivity by age group, with Wilson 95% confidence intervals. The Cochran–Armitage trend was positive but non-significant (*p* = 0.13).

Concomitant STI seropositivity increased monotonically across age groups, from 0.7% (95% CI 0.1–4.1) in patients aged 17–24 years to 3.5% (95% CI 1.6–7.4) in those ≥ 45 years (Table 5, Figure 3). The Cochran–Armitage test indicated a positive but non-significant trend (z = 1.50; *p* = 0.13), reflecting the limited number of seropositive cases. This age-related pattern may partly reflect the cumulative nature and persistence of some viral and treponemal serological markers.

### 3.6. Exploratory Demographic Associations

In exploratory univariable analysis (Table 6), anti-HBs negativity was significantly associated with concomitant STI seropositivity (OR = 5.63; 95% CI 1.60–19.83; *p* = 0.003). Age ≥ 30 years was also associated with seropositivity (OR = 3.73; 95% CI 1.08–12.93; *p* = 0.032), whereas male sex showed a positive but non-significant association (OR = 2.87; 95% CI 0.66–12.55; *p* = 0.184). A complementary Firth penalized multivariable model was directionally concordant and also showed a statistically significant association for anti-HBs negativity (OR = 3.64; 95% CI 1.08–12.28). Given the limited number of events, these findings are considered exploratory and hypothesis-generating.

### 3.7. Sensitivity Analysis of the Anti-HBs Association

Because anti-HBs negativity indicates susceptibility to HBV and HBsAg is a component of the composite outcome, the observed association could partly reflect their structural relationship. We therefore repeated the analysis after removing HBsAg from the outcome (Table 7). Of the eight HBsAg-positive patients, seven were anti-HBs-negative (anti-HBs untested in one). After excluding HBV, the association between anti-HBs negativity and the remaining STI markers (HCV, HIV, and syphilis) attenuated and was no longer statistically significant (OR = 2.73; 95% CI 0.70–10.69; *p* = 0.200) although the direction of effect was preserved.

### 3.8. Testing Availability and Comparison of Tested Versus Untested Patients

Because seroprevalence estimates depend on which patients happened to be tested, we compared the age and sex distribution of tested versus untested patients for each serological marker (Table 8). For every marker, tested and untested patients were statistically similar in age and sex (all *p* > 0.3, independent-samples *t*-test for age and χ^2^ test for sex), providing no evidence that testing was preferentially directed toward older, younger, male, or female patients. This does not rule out selection on unmeasured clinical factors (e.g., clinician suspicion of a specific infection), which could not be assessed retrospectively. The distribution of missing serological data for each marker is presented in Table 9.

## 4. Discussion

In this five-year cross-sectional study of 779 patients with AGW, the seroprevalence of concomitant STIs was low but non-negligible (2.4%; 95% CI 1.6–3.8). HBsAg occurred in 1.4% and anti-HCV in 0.9% of patients, anti-HIV was reactive in 0.2%, and treponemal antibody was reactive in 1.6% of those tested. To our knowledge, this is among the few two-centre, multi-year studies in Türkiye and the first to include the Central Black Sea region, extending previous single-centre reports [7,8,9,12,13] to a broader population. Seropositivity increased with age, and anti-HBs negativity emerged as the demographic factor most strongly associated with concomitant STI seropositivity. These estimates reflect serological evidence of infection with the tested pathogens only and should not be interpreted as the overall burden of concomitant STIs in this population, since Chlamydia trachomatis, Neisseria gonorrhoeae, herpes simplex virus, and Mycoplasma genitalium were not assessed.

### 4.1. Seroprevalence in Context

Our HBsAg estimate (1.4%) is lower than older Turkish community estimates (2.8–5.5%) [15,16] and is consistent with the marked decline in HBV prevalence among younger, vaccinated birth cohorts following the introduction of universal infant HBV immunization in Turkiye in 1998 [17]. The anti-HBs immunity rate (53.5%) is comparable to a recent Turkish AGW series [12]. The anti-HCV rate (0.9%) lies within the reported community range (0.3–2.1%) [7,9]. Anti-HIV was reactive in only one of 593 tested patients (0.2%; 95% CI 0.0–0.9), markedly lower than the 2.9% reported from a tertiary HIV-referral centre [8], likely reflecting our centre’s different catchment population. The detection of treponemal-reactive, non-treponemal-negative syphilis—interpreted via the reverse-sequence algorithm as compatible with current or previous syphilis infection; clinical stage and treatment status were unavailable—illustrates the value of serological screening in identifying otherwise silent infection. In similar studies, VDRL positivity rates were reported as 3.1% by Ünal et al. and 11.9% by Mueller et al., whereas no cases were detected in the studies by Aktaş and Sturgiss [9,13,18]. These findings reveal significant variability regarding reported syphilis serological markers in patients with anogenital warts; this variability may reflect differences in study populations, geographic regions, sample sizes, and syphilis testing algorithms. In our study, treponemal antibody reactivity was detected in 1.6% of the tested patients; however, as these tests evaluate different aspects of syphilis serology, this rate should not be directly compared with VDRL positivity rates.

### 4.2. Microbiological Interpretation of the Age and Anti-HBs Findings

The increase in seropositivity with age is biologically coherent, reflecting the cumulative, persistent nature of chronic viral and treponemal markers and the strong protection of younger, HBV-vaccinated cohorts [17]. The anti-HBs negativity association must be interpreted cautiously: because most HBsAg-positive patients (seven of the eight tested) were anti-HBs-negative, part of this association is structural, and our sensitivity analysis confirmed that it does not persist as an independent marker of non-HBV STIs. Its principal practical value is therefore in identifying patients lacking HBV protection, supporting HBV vaccination assessment in this population.

A further point concerns what each reactive marker can and cannot establish. HBsAg positivity indicates current HBV infection but does not by itself establish present infectivity; anti-HCV reactivity indicates prior exposure or possible current infection, which cannot be distinguished without HCV-RNA testing; and treponemal reactivity with a non-reactive non-treponemal test is consistent with prior exposure, but assigning a specific clinical stage (previously treated versus late/latent syphilis) requires clinical and treatment history that were not available to us. We have therefore avoided the terms “active” and “past or resolved infection” in describing these findings, reporting instead marker-specific results and the descriptive subgroup shown in Table 3. This distinction remains important for accurate counselling and for avoiding overinterpretation of composite seroprevalence estimates.

It is also important to assess whether increased access to the HPV vaccine during the study period influenced the epidemiological profile of patients presenting with AGW. HPV vaccination status was not recorded in our dataset for this period in Türkiye; therefore, this specific effect could not be directly evaluated. Nevertheless, the expansion of HPV vaccination practices may reduce the incidence of AGW over time, and it would be appropriate to consider this factor in future epidemiological studies [19]. Beyond its potential influence on the epidemiological profile of AGW presentations, HPV vaccination has direct relevance to the prevention of the concomitant STIs highlighted in this study. Quadrivalent and nonavalent HPV vaccines, which include HPV 6 and 11 antigens alongside high-risk oncogenic types, have been shown to substantially reduce anogenital wart incidence in vaccinated cohorts, and real-world surveillance following vaccine introduction has demonstrated marked declines in overall cervical and anogenital HPV prevalence, including among unvaccinated individuals through herd protection [20]. Because AGW and concomitant STIs share overlapping sexual transmission routes, as reflected in the co-infection patterns observed in our cohort, population-level HPV vaccination may plausibly reduce not only AGW incidence itself but also the downstream burden of concomitant STI screening triggered by AGW presentation. These considerations reinforce the public-health rationale for expanding HPV vaccination coverage in Türkiye, alongside the serological STI screening practices already recommended for patients presenting with AGW.

### 4.3. From Prediction to Exploration: A Deliberate Framing

We deliberately framed the demographic analysis as exploratory rather than as a clinical prediction rule. Even with 19 seropositive events in the expanded cohort, derivation of a validated risk score would risk overfitting and circular reasoning, and any apparent discriminative performance would be unstable. We therefore report associations with appropriate confidence intervals and penalized estimates, while refraining from proposing a clinical decision tool. The consistent direction of the anti-HBs association across univariable and penalized analyses nonetheless generates a testable hypothesis for adequately powered, multicentre studies.

## 5. Limitations

This study has several limitations. First, its retrospective design and the geographic proximity of the two participating centres (Amasya and Tokat, both in the Central Black Sea region) may still limit generalizability. Second, the number of seropositive events (*n* = 19) was limited, rendering the analyses exploratory and potentially subject to type II error. The panel covered routinely screened STIs only; herpes simplex virus, Neisseria gonorrhoeae, and Chlamydia trachomatis were not tested, so the true burden of concomitant STIs may be underestimated. Behavioural and sociodemographic risk factors (number of partners, condom use, prior STI history, and HIV pre-exposure prophylaxis [PrEP] use) were unavailable in the retrospective records and could not be incorporated into the risk-profile analyses. The cross-sectional, seroprevalence-based outcome combines markers whose current clinical significance differs (Section 3.4), which should be considered when interpreting the age association. The inclusion of two centres nonetheless modestly improves generalizability within this region compared with the original single-centre design; extrapolation to other regions or healthcare settings, particularly those with different STI epidemiology or HPV vaccination coverage, should still be made with caution. Finally, the annual patient counts (Figure 1) span calendar years of differing completeness—the study period begins in December 2020 and ends in October 2025—thus, year-to-year counts may partly reflect partial-year ascertainment, referral patterns, or changes in testing practice rather than a true increase in AGW incidence; these counts are therefore presented descriptively rather than as evidence of a temporal trend. Studies of concomitant bacterial STIs and anal HPV in other populations [21,22] illustrate the type of co-detection data our serology-based AGW cohort cannot provide; although not directly comparable, they help contextualize the likely unmeasured burden of bacterial STIs in our population and reinforce that causal inferences should not be drawn from co-detection alone.

## 6. Conclusions

Annual AGW presentations were characterized throughout the study period; however, due to the lack of strict comparability between observation periods, the annual figures were interpreted descriptively. Serological reactivity was detected among the tested STI markers, and variations were observed across age groups. Although the age-related increase in the frequency of serological reactivity was not statistically significant, a descriptive difference in distribution across age groups was noted. Exploratory analyses identified an association between anti-HBs negativity and the composite serological outcome; however, sensitivity analysis revealed that this association largely stemmed from the structural relationship with HBV status and did not support an independent association with non-HBV STI markers. These findings underscore the importance of considering HBV vaccination status during the clinical assessment of AGW patients and provide descriptive data on the distribution of concurrent serological findings in this patient population.

Nevertheless, given the study’s retrospective design, the incomplete and non-standardized administration of serological tests, and the limited number of reactive composite outcomes, the findings should be regarded as hypothesis-generating. Prospective, multicenter studies incorporating standardized serological testing, behavioural risk factors, and appropriate confirmatory assessments are required to further elucidate the clinical significance of reactive serological markers in AGW patients.

## Figures and Tables

**Table 1:** Demographic characteristics of patients with anogenital warts *(n* = 779).

Variable	Category	Value
Age, years	Mean ± SD (range)	35.80 ± 12.32 (17–87)
Age group	<30/≥30 years	316 (40.6%)/463 (59.4%)
Sex	Male/Female	585 (75%)/194 (25%)
Admission	Outpatient/Inpatient	288 (90.3%)/31 (9.7%) *n* = 319

SD, standard deviation.

**Table 2:** Seroprevalence of concomitant STIs and anti-HBs immunity, with Wilson 95% confidence intervals.

Serological Marker	Positive, *n*	Tested, *n*	Prevalence, %	95% CI
HBsAg	8	586	1.4	0.7–2.7
Anti-HCV	5	582	0.9	0.4–2.0
Anti-HIV	1	593	0.2	0.0–0.9
Treponemal Ab (anti-Tp)	7	446	1.6	0.8–3.2
VDRL/RPR	2	577	0.3	0.1–1.3
Anti-HBs (immune)	289	540	53.5	49.3–57.7

CI, confidence interval (Wilson score method); STI, sexually transmitted infection. The denominators reflect the number of patients tested for each marker. Anti-HBs reactivity denotes immunity and is reported separately from the composite outcome.

**Table 3:** The number of the five serological markers tested per patient.

Markers Tested, *n* (of 5)	Patients, *n* (%)
0	171 (22.0%)
1	2 (0.3%)
2	16 (2.1%)
3	31 (4.0%)
4	138 (17.7%)
5 (complete panel)	421 (54.0%)

Markers: HBsAg, anti-HCV, anti-HIV, VDRL/RPR, and treponemal antibody. The percentages are of the full cohort (*n* = 779).

**Table 4:** Reactive-marker subgroups by inferred clinical relevance.

Outcome Definition	*n*	Prevalence, %	95% CI
HBsAg-positive, anti-HIV-reactive, or dual treponemal + non-treponemal reactivity (staging not confirmed)	11	1.4	0.8–2.5
Any reactive serological marker (clinical significance not further classified)	19	2.4	1.6–3.8

**Table 5:** Concomitant STI seropositivity by age group.

Age Group, Years	Patients, *n*	Seropositive, *n*	Prevalence, %	95% CI
17–24	135	1	0.7	0.1–4.1
25–34	297	7	2.4	1.1–4.8
35–44	174	5	2.9	1.2–6.5
≥45	173	6	3.5	1.6–7.4

Cochran–Armitage trend test: z = 1.50, *p* = 0.13. CI, confidence interval (Wilson score method).

**Table 6:** Exploratory associations of demographic factors with concomitant STI seropositivity.

Factor	Concomitant STI Seropositivity Present, *n*	Concomitant STI Seropositivity Absent *n*	OR (95% CI)	*p*
Anti-HBs negative	14	237	5.63 (1.60–19.83)	0.003
Anti-HBs positive (reference)	3	286	1.00 (reference)	—
Anti-HBs missing	2	237	—	—
Age ≥ 30 years	16	447	3.73 (1.08–12.93)	0.032
Age < 30 years (reference)	3	313	1.00 (reference)	—
Male sex	17	568	2.87 (0.66–12.55)	0.184
Female sex (reference)	2	192	1.00 (reference)	—

Abbreviations: OR, odds ratio; CI, confidence interval. ORs were calculated using Fisher’s exact test with Woolf–Haldane-corrected logit 95% CIs, with each level compared with the stated reference category. The columns “Concomitant STI seropositivity +, *n*” and “Concomitant STI seropositivity −, *n*” indicate the numbers of patients with and without composite concomitant STI seropositivity, respectively, within each factor level. Patients with missing data for the corresponding factor were excluded from the OR calculation and are shown separately where applicable. Analyses are exploratory.

**Table 7:** Sensitivity analysis: the association of anti-HBs negativity with the composite STI outcome, with and without HBV markers.

Outcome Definition	Events, *n*	OR (95% CI)	*p*
Any STI incl. HBV (HBsAg)	19	5.63 (1.60–19.83)	0.003
STI excl. HBV (HCV, HIV, syphilis)	11	2.73 (0.70–10.69)	0.200

OR, odds ratio (Fisher’s exact test). Of the 8 HBsAg-positive patients, 7 were anti-HBs-negative (anti-HBs untested in 1).

**Table 8:** Demographic characteristics of patients tested versus not tested, by serological marker.

Marker	Tested, *n*	Tested: Age, Mean ± SD (%Male)	Untested, *n*	Untested: Age, Mean ± SD (%Male)	*p* (Age/Sex)
HBsAg	586	35.68 ± 12.27 (74.6%)	193	36.15 ± 12.48 (76.7%)	0.655/0.623
Anti-HCV	582	35.58 ± 12.14 (75.1%)	197	36.44 ± 12.83 (75.1%)	0.414/1.000
Anti-HIV	593	35.55 ± 12.17 (74.9%)	186	36.60 ± 12.79 (75.8%)	0.324/0.873
VDRL/RPR	577	35.59 ± 12.18 (76.3%)	202	36.39 ± 12.70 (71.8%)	0.437/0.242
Treponemal Ab (anti-Tp)	446	35.95 ± 12.42 (76.5%)	333	35.60 ± 12.19 (73.3%)	0.694/0.351

SD, standard deviation. *p* values from independent-samples *t*-test (age) and χ^2^ test (sex), comparing tested and untested patients for each marker.

**Table 9:** Missing serological data by marker.

Serological Marker	Tested, *n*	Missing, *n*	Missing, %
HBsAg	586	193	24.8
Anti-HCV	582	197	25.3
Anti-HIV	593	186	23.9
VDRL/RPR	577	202	25.9
Treponemal Ab (anti-Tp)	446	333	42.7
Anti-HBs	540	239	30.7

The denominator for all markers is the full cohort (*n* = 779). Anti-HBs is analyzed separately as a marker of immunity and is not part of the composite STI outcome.

## Data Availability

The raw data supporting the conclusions of this article will be made available by the corresponding author on request.

## Supplementary Materials

The following supporting information can be downloaded at: https://www.mdpi.com/article/10.3390/pathogens15101062/s1, File S1: STROBE checklist: completed checklist for the present study.
