# Supplementary material for: Serological Markers of Selected Sexually Transmitted Infections (HBV, HCV, HIV, and Syphilis) in Patients with Anogenital Warts: A Two-Centre, Five-Year Study
Source: Pathogens. 2026 Oct 8;15(10):1062. doi: 10.3390/pathogens15101062 (PMC13708646; doi:10.3390/pathogens15101062)
Supplement: Supplementary file 1 [file pathogens-15-01062-s001.zip › pathogens-4442100-supplementary.pdf]

File S1: STROBE checklist: completed checklist for the present study

Section	Item	Recommendation	Reported in manuscript (section/table)
**Title and abstract**	1a	Indicate the study's design with a commonly used term in the title or the abstract	Title: "...A Two-Centre, Five-Year Study"; Abstract Methods: "retrospective, cross-sectional study"
	1b	Provide an informative and balanced summary of what was done and what was found	Abstract (Background/Methods/Results/Conclusion structure)
**Introduction**	2	Explain the scientific background and rationale for the investigation being reported	Section 1, Introduction, paragraphs 1–4
	3	State specific objectives, including any prespecified hypotheses	Section 1, final paragraph (primary objective and exploratory secondary objective stated)
**Methods**	4	Present key elements of study design early in the paper	Section 2.1, first sentence
	5	Describe the setting, locations, and relevant dates, including periods of recruitment, exposure, follow-up, and data collection	Section 2.1: two centres (Amasya, Tokat), December 2020–October 2025
	6a	Give the eligibility criteria, and the sources and methods of selection of participants	Section 2.1: inclusion (clinical AGW diagnosis, age ≥17y) and exclusion (missing age/sex, diagnosis date outside period) criteria; two extraction methods described
	6b	Give the eligibility criteria, and the sources and methods of case ascertainment and control selection. Give the rationale for the choice of cases and controls (N/A — not a case-control study)	Not applicable (cross-sectional design, no case/control selection)

	7	Clearly define all outcomes, exposures, predictors, potential confounders, and effect modifiers. Give diagnostic criteria, if applicable	Section 2.3 (Definitions); Section 2.4 (demographic factors examined)
	8	For each variable of interest, give sources of data and details of methods of assessment (measurement). Describe comparability of assessment methods if there is more than one group	Section 2.2 (Microbiological methods); Section 2.1 (two data-extraction routes described, with their differences and limitations noted in Section 5)
	9	Describe any efforts to address potential sources of bias	Section 5 (Limitations): selective/incomplete testing, non-uniform syphilis algorithm, unverifiable test–diagnosis interval, all explicitly discussed; Section 3.8 (Table 7) tests for demographic selection bias in who was tested
	10	Explain how the study size was arrived at	Section 2.1: All consecutive patients meeting the predefined eligibility criteria during the study period were included; therefore, no a priori sample-size calculation or formal power analysis was performed. The final sample comprised 779 eligible patients.
	11	Explain how quantitative variables were handled in the analyses. If applicable, describe which groupings were chosen and why	Section 2.4: continuous vs categorical handling; age dichotomised at ≥30 years and grouped into four bands (17–24, 25–34, 35–44, ≥45) for trend analysis (Table 4)
	12a	Describe all statistical methods, including those used to control for confounding	Section 2.4, in full
	12b	Describe any methods used to examine subgroups and interactions	Section 2.4 (age-group trend, sensitivity analysis excluding HBV markers); Section 3.8 (tested vs untested comparison)

	12c	Explain how missing data were addressed	Section 2.4 (marker-specific denominators); Section 3.2/3.8 and Tables 2b, 7, 8 (testing completeness, demographic comparison, missing-data percentages)
	12d	If applicable, describe analytical methods taking account of sampling strategy (N/A — no complex sampling design)	Not applicable
	12e	Describe any sensitivity analyses	Section 3.2 (complete-case composite prevalence); Section 3.7 (sensitivity analysis excluding HBV markers)
**Results**	13a	Report the numbers of individuals at each stage of the study (e.g., numbers potentially eligible, examined for eligibility, confirmed eligible, included in the study, completing follow-up, and analysed)	Section 3.1 (n=779 included); Table 2b (testing completeness per patient); Section 3.1: 779 eligible patients were included. All consecutive eligible patients identified from the two source systems during the study period were included. Because this was a retrospective study based on existing records, a formal participant flow diagram showing every screening/exclusion step could not be reconstructed. Testing completeness is additionally detailed in Table 2b.
	13b	Give reasons for non-participation at each stage	Section 2.1 (exclusion criteria); reasons for missing serological results are structural (routine clinical ordering, not study-driven non-participation)
	13c	Consider use of a flow diagram	Not applicable / A formal participant flow diagram was not constructed because all consecutive eligible patients were included and the exact number of records screened and excluded at each retrospective extraction step could not be reconstructed.
	14a	Give characteristics of study participants (e.g., demographic, clinical, social) and information on exposures and potential confounders	Table 1; Section 3.1
	14b	Indicate the number of participants with missing data for each variable of interest	Table 8 (missing data by marker); Table 2 ('Tested, n' column for every marker)

	14c	Summarise follow-up time (e.g., average and total amount) (N/A — cross-sectional design, no follow-up)	Not applicable
	15	Report numbers of outcome events or summary measures	Table 2 (per-marker positives); Table 3 (composite outcome subgroups); Section 3.2–3.4
	16a	Give unadjusted estimates and, if applicable, confounder-adjusted estimates and their precision (e.g., 95% confidence interval). Make clear which confounders were adjusted for and why they were included	Table 5 (univariable, Woolf/Fisher); Section 3.6 (Firth-penalised multivariable model; all three covariates included a priori, not selected by significance)
	16b	Report category boundaries when continuous variables were categorized	Section 2.4 and Table 4 (age-group boundaries stated)
	16c	If relevant, consider translating estimates of relative risk into absolute risk for a meaningful time period (N/A — cross-sectional prevalence study, no time-period risk)	Not applicable
	17	Report other analyses done (e.g., analyses of subgroups and interactions, sensitivity analyses)	Table 6 (sensitivity analysis excluding HBV); Table 7 (tested vs untested comparison); Section 3.4 (marker-subgroup analysis)
**Discussion**	18	Summarise key results with reference to study objectives	Section 4, first paragraph
	19	Discuss limitations of the study, taking into account sources of potential bias or imprecision. Discuss both direction and	Section 5 (Limitations), in full — six numbered points covering generalisability, statistical power, testing completeness/bias, panel scope, unmeasured behavioural factors, and temporal-trend caveats

		magnitude of any potential bias	
	20	Give a cautious overall interpretation of results considering objectives, limitations, multiplicity of analyses, results from similar studies, and other relevant evidence	Section 4.1–4.3, drawing comparisons with cited Turkish and international literature
	21	Discuss the generalisability (external validity) of the study results	Section 5 (two-centre design and regional homogeneity explicitly discussed)
**Other information**	22	Give the source of funding and the role of the funders for the present study and, if applicable, for the original study on which the present article is based	Funding statement: “None”
